# Report on Last Ovariotomy Performed by Prof. J. P. White

**Published:** 1881-11

**Authors:** C. M. Daniels


### Clinical Reports.

### Report on Last Ovariotomy Performed by Prof. J. P. White.

By C. M. Daniels, M. D.

Mrs. D., aged 52, commenced first to show signs of enlargement twelve years ago. For past few months had increased more rapidly, prostration becoming such that life was endangered.

Ovarian tumor diagnosed by the two attending physicians, but doubt was entertained by several in previous consultation. Dr. White first saw the case Sept. 7th, and at once confirmed the diagnosis and proceeded to operate. Patient under ether, exploratory incision in median line three inches below umbilicus, and there was considerable uncertainty as to where the aponeurosis ended and tumor sack commenced, it was so perfectly agglutinated to the abdominal parietes. The sack was finally opened with the point of a scalpel and about five quarts seropurulent fluid escaped. Abdominal opening being enlarged to about four inches and then began the difficult operation of separating the adhesions. In fact it was with nearly as much difficulty as it would have been to separate the abdominal muscles and the emptied sack after removal seemed almost as firm and tough as moistened leather, very great force being necessary to accomplish its removal, although there were no intestinal adhesions. The tumor was a monocyst. A small piece of omentum was removed. No general hemorrhage. The pedicle was ligated with extra strong carbolized silk and returned to abdominal cavity, which was then thoroughly sponged. The opening was closed in the usual manner, with silver wire for the deep, and silk for superficial sutures. Carbolized compress and long adhesive straps completed the dressing. Patient was very much exhausted and the prognosis seemed unfavorable. Dr. White remarked that it was the most difficult operation of the kind he ever performed, although time consumed was only forty minutes. The contents of the sack were rapidly disintegrating, and doubtless patient could have survived but a short time.

A report from the attending physicians six weeks after operation is as follows: For twenty-four hours the extreme prostration continued, the pain was severe until controlled by morphia, and stimulants were given freely. During the first five days the pulse ranged from 120 to 160; temperature being 102° to 105°, after that time there was a gradual improvement. At the end of a week the dressing and superficial sutures were removed, discharged pus seemed healthy and so continued. The edges of the wound united except at superior angle, and a small fistula still persists. At ten days the deep sutures were removed and union was perfect. There was some nausea during the first week, which was easily controlled by ordinary remedies. Bowels and bladder soon resumed their normal functions and convalescence seems now fully established. Thus, did the last as well as the first ovariotomy performed by Prof. Jas. P. White prove a success.

